# Effect of PARACT (PARAmedical Interventions on Patient ACTivation) on the Cancer Care Pathway: Protocol for Implementation of the Patient Activation Measure-13 Item (PAM-13) Version

**DOI:** 10.2196/17485

**Published:** 2020-12-08

**Authors:** Elise Verot, Wafa Bouleftour, Corinne Macron, Romain Rivoirard, Franck Chauvin

**Affiliations:** 1 Centre Hygée University of Saint-Etienne University of Lyon Saint-Priest-en-Jarez France; 2 Medical Oncology Department Institut de Cancérologie de la Loire Saint-Priest-en-Jarez France; 3 Institut de Cancérologie de la Loire Saint-Priest-en-Jarez France

**Keywords:** oncology, nursing, implementation science, PAM-13, patient activation, REALM-R, health literacy, mixed method

## Abstract

**Background:**

The increase in the number of cancer cases and the evolution of cancer care management have become a significant problem for the French health care system, thereby making patient empowerment as a long sought-after goal in chronic pathologies. The implementation of an activation measure via the Patient Activation Measure-13 item (PAM-13) in the course of cancer care can potentially highlight the patient’s needs, with nursing care adapting accordingly.

**Objective:**

The objectives of this PARACT (PARAmedical Interventions on Patient ACTivation) multicentric study were as follows: (1) evaluate the implementation of PAM-13 in oncology nursing practices in 5 comprehensive cancer centers, (2) identify the obstacles and facilitators to the implementation of PAM-13, and (3) produce recommendations for the dissemination of such interventions in other comprehensive cancer centers.

**Methods:**

This study will follow the “Reach, Effectiveness, Adoption, Implementation, and Maintenance” framework and will consist of 3 stages. First, a robust preimplementation analysis will be conducted using the Theoretical Domains Framework (TDF) linked to the “Capability, Opportunity, Motivation, and Behavior” model to identify the obstacles and facilitators to implementing new nursing practices in each context. Then, using the Behavior Change Wheel, we will personalize a strategy for implementing the PAM-13, depending on the specificities of each context, to encourage acceptability by the nursing staff involved in the project. This analysis will be performed via a qualitative study through semistructured interviews. Second, the patient will be included in the study for 12 months, during which the patient care pathway will be studied, particularly to collect all relevant contacts of oncology nurses and other health professionals involved in the pathway. The axes of nursing care will also be collected. The primary goal is to implement PAM-13. Secondary factors to be measured are the patient’s anxiety level, quality of life, and health literacy level. The oncology nurses will be responsible for completing the questionnaires when the patient is at the hospital for his/her intravenous chemotherapy/immunotherapy treatment. The questionnaires will be completed thrice in a year: (1) at the time of the patient’s enrollment, (2) at 6 months, and (3) at 12 months. Third, a postimplementation analysis will be performed through semistructured interviews using the TDF to investigate the implementation problems at each site.

**Results:**

This study was supported by a grant from the French Ministry of Health (PHRIP PARACT 2016-0405) and the Lucien Neuwirth Institute of Cancerology of Saint-Etienne, France. Data collection for this study is ongoing.

**Conclusions:**

This study would improve the implemented targeted nursing interventions in cancer centers so that a patient is offered a personalized cancer care pathway. Furthermore, measuring the level of activation and the implementation of measures intended to increase such activation could constitute a significant advantage in reducing social health inequalities.

**Trial Registration:**

ClinicalTrials.gov NCT03240341; https://clinicaltrials.gov/ct2/show/NCT03240341

**International Registered Report Identifier (IRRID):**

DERR1-10.2196/17485

## Introduction

### Background

The increase in the number of cancer cases and the evolution of cancer care management have been generating a shift toward chronicity since many years. This phenomenon poses a significant problem for the French health care system [1,2]. As the number of patients treated as outpatients increases, the duration of hospitalization decreases and the care pathways become more complex [1,3,4]. The first 2 French cancer plans have given a structure to the field of cancer care in the domains of both treatment and research [5,6]. The objective of the 2014-2019 French cancer plan was to improve the interfaces between the different fields of interventions for a smoother transfer of innovation and for more fluidity in cancer care pathways [1]. However, the French health care system is centered on acute care whether it involves the management of benign acute diseases combining simple technical procedures or a multitude of complex interventions to treat the severity of acute diseases [7]. One can therefore comprehend the difficulty of integrating chronic care into hospitals in terms of its organization and due to certain attitudes adopted by caregivers. Three types of interventions were identified as part of the research conducted within our health services and performance research team, the objective of which is the performance of the provision of care and the performance of the patient pathway: (1) intervention to improve patient navigation (patient navigation) [8], (2) intervention to support and improve health literacy [9,10], and (3) intervention to support and improve patient empowerment during care (patient empowerment) [11]. These 3 types of interventions are considered to be evidence-based since they have been assessed in an experiential or quasi-experiential context to optimize the care pathway.

### Types of Interventions

#### Patient Navigation

According to the patient navigation experience developed by Harold Freeman in 1990 in Harlem for breast cancer screening, the aim of “Nurse Navigation” in the United States or “Pivot Nurses” in Canada is to better guide patients through a complex care pathway [12,13]. The principle of navigation serves to eliminate barriers having an adverse effect on the quality of care in all phases of prevention, detection, treatment, and posttreatment [14-17]. In France, patient navigation was implemented in oncology clinics with the national initiative of cancer coordination nurses in accordance with the 2009-2013 French Cancer Plan [17]. However, not all French hospitals employed navigation nurses.

#### Health Literacy

The term “health literacy” is based on the definition given by Sørensen, since it is an accepted reference at the European level [18]. She defines health literacy as “the knowledge, skills, motivation, and ability of an individual to identify, understand, evaluate, and use health information when making decisions in the health care, disease prevention, and health promotion contexts to maintain or improve the quality of life” [18]. It has been established that doctors often communicate ineffectively with their patients by providing them with explanations that are theoretically beyond their comprehension, leading to a relatively low health literacy. The most affected areas include the general context, explanations of the patient’s state of health, and treatment methods [19]. Research has objectively demonstrated that when doctors provide their patients with useful health information while also responding to patients’ emotions, the latter express a greater sense of control and hope, which has a direct positive impact on their quality of life as well as on their chances of survival [20]. It therefore seems evident that clear communication adapted to the patient is essential in the management of care for patients with cancer. After all, patients must assimilate a sum of complex explanations in order to make informed decisions about their treatment options and management of their symptoms [21]. Specifically, in oncology clinics, being mindful of patients’ needs in terms of health literacy is an important aspect of treatment since it results in care that is both focused and safe [22]. Furthermore, various studies indicate that a low level of health literacy can have an impact on the following parameters [23,24]: (1) readmission and recourse to hospitalization, (2) problems understanding medical prescriptions, (3) increase in adverse effects, (4) less awareness regarding prevention, (5) higher prevalence of health risk factors, (6) low autonomy in chronic disease management, (7) poor health outcomes, (8) less effective communication with health care professionals, (9) increased health care costs, and (10) poor general health and increased mortality.

The REALM-R (rapid estimate of adult literacy in medicine-revised) is a health literacy test that was developed to evaluate patients in a clinical setting [25]. Bass et al provided evidence to support the validity and reliability of a shortened version of the REALM [26]. Grandjacquot recently adapted it in the French language and the test proved effective in detecting inadequate levels of health literacy [27].

#### Patient Empowerment

This process was developed in the United States of America and Canada with the concept of activation and has not yet been widely applied in France. It is defined as a process of knowledge acquisition, skill development, and self-esteem development, thereby enabling the patient to be an actor or to engage in a proactive manner for his or her health care [28,29]. The Patient Activation Measure (PAM) was developed by Hibbard et al ﻿to assess knowledge, skills, and confidence in managing health [30,31]. This questionnaire can be used at different times of care [31,32]. The PAM-13 item (PAM-13) is the shortened version of the PAM. It is actually used in several contexts of disease and in many countries around the world [31,33-39]. In particular, Prey et al have demonstrated its reliability and validity while using it with inpatient individuals in oncology clinics [40]. The PAM-13 is used to determine the patient’s score and activation level [41]. Four activation levels are defined: “believes active role important” (level 1), “confidence and knowledge to take action” (level 2), “taking action” (level 3), and “staying the course under stress” (level 4). A validated French version is available (Multimedia Appendix 1).

In general, the lower the level, the more passive the patients behave in regard to their health; the higher the level, as measured by the PAM-13, the more proactive patients are with regard to their health. In all probability, the latter group would commit to improving their health behaviors [38,42]. The importance of the patient’s role in self-management of chronic illness, including making daily decisions about treatment, physical activity, or diet, has been increasingly recognized [43,44]. A high activation level can result in improved health-friendly behaviors [45], appropriate use of the health care system [42,46], a critical perspective and participation in decision-making processes with stakeholders in the health care system [42], improved management [47] and better control of chronic diseases [48,49], and a reduction in health care costs [50]. Patients with a high activation level generally manifest less misguided practices of care and better treatment adherence [46,51]. This concept of commitment can be initiated and applied to treatment and to the management of services and establishments or health policies [52].

### Objective of This Study

We believe that effective action in cancer care requires progressive patient empowerment. Several studies have illustrated the role doctors play in the patient activation process [53-56]. However, nearly all such studies have focused on basic care, with the general practitioner positioned as the “gatekeeper.” Hospitals and their staff can contribute to improving health-related behaviors and mobilizing their patients [57,58]. Nevertheless, in comprehensive cancer centers, the modulation of activation is likely varied and more complex because these facilities differ in terms of the specificities of nursing specialties they offer. Nursing positions and the provision of supportive care in oncology clinics can vary greatly depending on the hospital; for example, there are nurse navigators, nurse educators, dieticians, oncopsychologists, osteopaths, physiotherapists, hypnotherapists, and nurses specialized in tobacco cessation. We can suppose that the patients’ activation is therefore largely modulated by the type of nurse and supportive care implemented within the hospital. Therefore, we can also assume that these varying cancer care pathways also lead to different results when considering vulnerabilities, access to specific measures, quality of life, and, consequently, patient activation. The World Health Organization has stated that although certain patients will be able to mobilize their own resources and succeed in coping with the situation, thereby eventually becoming (pro)active, others will need to be guided by their caregivers [59]. The implementation of an activation measure via the PAM-13 by navigation nurses or other registered nurses would highlight the patients’ needs [60].

We are aware of the need to perform a periodic screening to identify new patient needs that emerge over time and adapt nursing measures to these needs that arise in the course of the cancer care pathway [61,62]. In addition, van Houtum et al suggest that patient activation is not a stable parameter in patients with chronic disease [63]. Greene et al provide a longitudinal measurement of PAM in order to better understand the timing of the activation process [41]. In France, the first results of the Observatory on Patient Expectations of the Unicancer Group, and more recently, findings of the new 2014-2019 Cancer Plan III (Objectives 2 and 7), underlined the need to shift current practices of care toward a more holistic view of the patient with cancer [1].

The PARACT (PARAmedical Interventions on Patient ACTivation) multicentric study aims to (1) evaluate the implementation of PAM-13 in oncology nursing practices in 5 comprehensive cancer centers, (2) identify the obstacles and facilitators to the implementation of PAM-13 at the different sites, and (3) produce recommendations for the dissemination of the intervention in other comprehensive cancer centers.

## Methods

### Implementation Study

**Figure 1:**
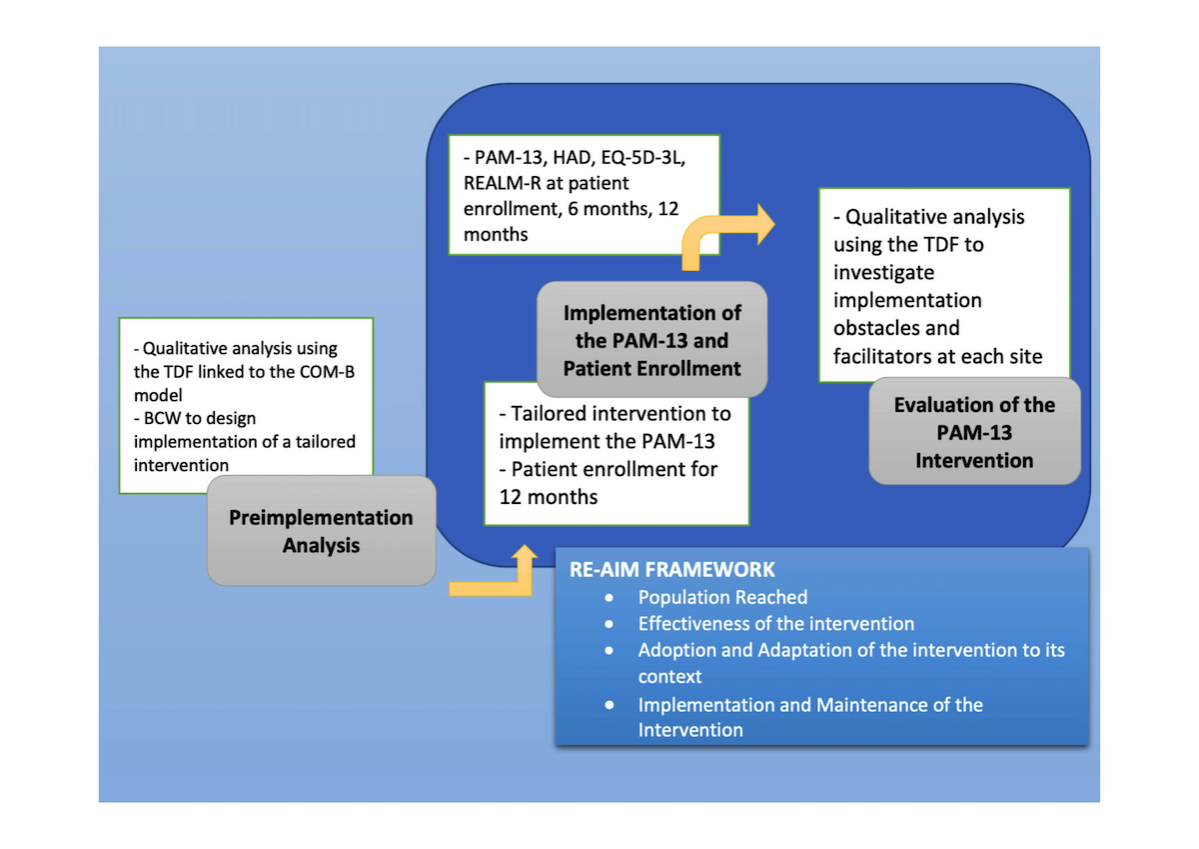
Overall design of the PARACT (PARAmedical Interventions on Patient ACTivation) multicentric study.

This is an implementation multicentric study. The PARACT study is part of the realistic evaluation of an intervention as described by Pawson and Tilley following Campbell’s work [64]. This approach favors multisite and multidisciplinary assessments to study the interactions of context-mechanism-outcomes configurations. This type of evaluation makes it possible to adapt the interventions according to the results observed, while considering the context. The assessment selected for this study will therefore be multidisciplinary, combining public health teams and human and social sciences teams. The aspect of navigation in establishments will be assessed in accordance with current recommendations [65]. The patient will act as his/her own control. Given that we are conducting a study of an implementation that is complex, the study will follow the RE-AIM (Reach, Effectiveness, Adoption, Implementation, and Maintenance) framework. The implementation process will consist of 3 stages (Figure 1).

1. First, a robust preimplementation analysis will be performed by using the Theoretical Domains Framework (TDF) linked to the COM-B (Capability, Opportunity, Motivation, and Behavior) model to identify the obstacles and facilitators to implementing new nursing practices in each context. Then, using the Behavior Change Wheel, a strategy for implementing the PAM-13 will be personalized, depending on the specificities of each context, in order to encourage acceptability by the nursing staff involved in the project. This preimplementation analysis will be carried out via a qualitative study through semistructured interviews.
2. Once this step is complete, the second step of enrolling patients in the study can begin. The patient will be included in the study for a period of 12 months. During his or her involvement, the patient care pathway will be studied, particularly to collect all relevant contacts of oncology nurses and other health professionals involved in the cancer care pathway. The axes of nursing care will also be collected. The primary goal is PAM-13. Secondary factors to be measured are the patient’s level of anxiety according to the Hospital Anxiety and Depression (HAD) scale, the assessment of patients’ quality of life as measured by the European Quality of Life-5 Dimensions-3 Level (EQ-5D-3L) questionnaire, and the level of health literacy using the REALM-R. The oncology nurses in charge of treating the patient in the cancer care pathway will be responsible for completing each of the questionnaires when the patient is at the hospital for his/her intravenous chemotherapy or immunotherapy treatment. The questionnaires will be completed 3 times in the course of 1 year: (1) at the time of the patient’s enrollment in the study, (2) at 6 months, and (3) at 12 months.
3. Finally, at the end of the study period and patient follow-up, a postimplementation analysis will be performed through semistructured interviews, using the TDF to probe implementation problems at each site. Four implementation elements will be measured within the framework of RE-AIM: reach the population (R), effectiveness of the intervention (E), adoption and adaptation of the intervention to its context (A), and maintenance of the intervention (M) [66].

### Research Design

#### Step 1: Preimplementation Analysis

Qualitative data will be collected in the form of individual semistructured interviews, with oncology nurses involved in setting up and conducting PAMs in different comprehensive cancer centers. This qualitative phase will take place before implementation. According to Malterud, qualitative research offers strong potential to understand a problem in all its dimensions from all angles and to study a phenomenon not yet documented. In addition, the production of qualitative data is essential to be able to question the caregivers’ representations [67]. It will enable us to assess the evolution of the perceptions of the nurses’ teams concerned in this study, the activation of the patients, the perception of their own roles, and their perceived and actual implications in this project. Ten semistructured interviews will be scheduled at each center. The special feature of TDF is that it specifically questions individual motivation and capacity factors, in addition to taking into account the physical and social environment of the context concerned by the implementation [68]. We will perform a qualitative analysis based on TDF to (1) build the semistructured interview grid [68,69], (2) conduct a qualitative analysis [70], (3) diagnose obstacles and facilitators to implementation [68], (4) draw up the intervention to ensure better acceptability, linking the TDF to the COM-B model and then to the Behavior Change Wheel [69].

Qualitative data will be analyzed in order to generate themes, that, in poststudy findings, can be associated with the TDF [68,69,71-74]. Specifically, we will apply the methodology framework proposed by Braun and Clarke [75].

1. Familiarization with the data: This phase involves reading and rereading the data to become immersed and intimately familiar with its content.
2. Generating initial codes: This phase involves generating succinct codes that identify important features of the data that might be relevant to answering our research question. It involves coding the entire data set, and after that, collating all the codes and all relevant data extracts together for later stages of analysis. Nvivo 11 pro software (QSR International) will be used to perform the analyses.
3. Searching for themes: The collection of codes will be worked on in pairs with a PhD researcher in sociology to validate a comprehensive interpretation and a grouping of code elements into themes.
4. The resulting themes will be compared to the TDF domains, independently by 2 researchers, and then compared and discussed. Items that could not be included in the TDF framework will be thematized separately.
5. Finally, a more refined coding will be used for each part of the verbatim used to illustrate the themes.

The COREQ (consolidated criteria for reporting qualitative research) will guide the reporting of the qualitative analysis [76].

#### Step 2: Implementation of the PAM-13 and Patient Enrollment

##### Number of Subjects Needed

As this is an implementation study, it is not possible to calculate a sample size required to respond to an efficacy hypothesis. The goal is to observe how many patients will be involved in the measurement of PAM-13 in real life. It is therefore not possible at this juncture to determine a set number of patients to be reached before ending the study.

Nevertheless, in order to stay within the confines of the study budget, it seems reasonable to limit the number of enrollments to approximately 150 patients per center in order to have a sufficient understanding of the implementation of the PAM-13 in the different facilities. A total of up to 600 patients will be included in the 5-site study (up to 150 patients per site). Two establishments will include a maximum of 75 patients per site.

We will implement the PAM-13 at each center and the study may begin enrolling patients, as outlined below.

##### Screening and Inclusion

The oncologists, and, as a priority, the cancer nurse navigator (if the establishment employs one) or an oncology care nurse involved in the announcement of the diagnosis, and finally, a registered nurse at the choice of the investigator of the different investigative centers will suggest patients who meet the inclusion criteria to participate in the PARACT study. First, the procedure and aim of the study will be explained to the patient, and then, he or she will be given a consent form to sign. Patients will have a period of reflection of at least half an hour—seemingly sufficient—considering the extremely low risk factor of participating in the PARACT study. The patients’ inclusion must be completed within 15 days of the consultation to announce the cancer pathology. The patient will be included in the study for a period of 12 months. During his or her involvement, the patient care pathway will be studied, particularly to collect all relevant contacts of oncology nurses and other health professionals involved in the cancer care pathway. Axes of nursing care will also be collected. The primary goal is PAM-13. Secondary factors to be measured are the patient’s level of anxiety according to the HAD scale, the assessment of patients’ quality of life as measured by the EQ-5D-3L questionnaire, and the level of health literacy by using the REALM-R screening instrument. The oncology nurses in charge of treating the patient in the cancer care pathway will be responsible for completing each of the questionnaires when the patient is at the hospital for his/her intravenous chemotherapy or immunotherapy treatment. The questionnaires will be completed 3 times in the course of 1 year: (1) at the time of the patient’s enrollment in the study, (2) at 6 months, and (3) at 12 months.

##### Patient Inclusion Criteria

The inclusion criteria for this study are as follows. The patient must be at least 18 years of age upon enrollment. The patient with cancer must have an estimated life expectancy of at least one year. The patient must be undergoing treatment involving intravenous chemotherapy or immunotherapy for any cancer or both of them as outlined in the decision of the multidisciplinary concertation meeting. The patient must be affiliated with a health insurance policy or entitled to social security coverage.

##### Patient Exclusion Criteria

Patients are excluded from this study for any of the following reasons. The patient has declined to participate. The adult patient is protected under legal guardianship or curatorship. The patient is unable to understand the procedure of the study. The patient has presented prior documentation indicating cognitive or psychiatric disorders. The patient does not understand the French language.

##### Validity and Reliability of the Questionnaires

The PAM-13 was developed by Hibbard et al to assess knowledge, skills, and confidence in managing health [30,31,77]. It is used in several contexts of disease and in many countries around the world [31,33-39]. Specifically, Prey et al have demonstrated its reliability and validity with inpatient individuals in oncology [40]. The validated French version will be used. The HAD scale will be used to measure the evolution of patients’ anxiety [78]. The United Kingdom National Health Service and French National Authority for Health recommend its use to assess anxiety and depression among inpatient individuals. Its reliability and validity are widely recognized [78-80]. The EQ-5D-3L is a validated tool for measuring the quality of life [81]. Several studies have been published and have provided evidence to support the validity and reliability of the EQ-5D in studies of cancer [82]. This self-questionnaire explores 5 dimensions: mobility, self-care, usual activities, pain/discomfort, and worry/pressure. The REALM-R test validated in French will be used to measure the level of patients’ health literacy. Bass et al provided evidence to support the validity and reliability of the shortened version of the REALM, which is named as REALM-R [26]. Grandjacquot recently adapted it in the French language, and it proved effective in detecting inadequate levels of health literacy [27].

#### Step 3: Evaluation of the Implementation

First, at the end of the inclusion period and patient follow-up, a postimplementation analysis through semistructured interviews using the TDF to investigate implementation obstacles and facilitators at each site will be performed. The same sample and qualitative analysis methodology as that relating to the preimplementation analysis will be used.

Second, 4 implementation outcomes will be measured. These outcomes would have been collected over the entire study by using the RE-AIM [66]. See overview of the implementation outcomes from the RE-AIM (Multimedia Appendix 1).

### Statistical Analysis

#### Design

The analyses will be performed using R 3.2.5 (R Foundation [83]) software. The characteristics of the patients will be described using the following statistics: for quantitative variables, number of available data, means, standard deviation, median, quartile 1, and quartile 3; and for qualitative variables, absolute and relative frequencies (expressed as percentage). To calculate patient activation, the guidelines provided by Insignia Health will be followed [84]. Activation will be measured quantitatively at enrollment, at 6 months, and at 12 months. The difference will be calculated between the baseline measurement and the 12-month measurement for all patients at each center. The mean of this difference will be compared between the comprehensive cancer center that employs a nurse navigator and each of the 4 other facilities. Activation will also be compared according to its evolution during the 12 months of follow-up by using a mixed linear model. The variables influencing the difference in the activation score will be included in this model. The search for factors influencing the activation level will be carried out using the following methods:

1. Initially, univariate analysis with chi-squared tests (or Fisher exact if the conditions are not met) for qualitative variables and Student two-sided *t* test (or Wilcoxon test if the conditions are not met) for quantitative variables will be performed.
2. Subsequently, multivariate analysis with all exploratory variables with a significance threshold lower than 0.2 will be introduced into the multivariate model. The interactions between the variables will be ensured in advance. If an interaction is detected, the choice of the variable to be introduced will be made based on clinical relevance. The choice of the best multivariate model will be made using a selective downward procedure on the Akaike information criteria.
3. Health literacy will be assessed via the score obtained by the REALM-R (a score <6 demonstrating a low health literacy score). The before/after health literacy increase will be assessed using a McNemar chi-square test using the score qualitatively (threshold at 6). A logistic regression model with random effect will be used in the multivariate analysis to eliminate any confounding factors with the same methodology as described above. The same methodology will be applied to the HAD scale (threshold at 11). The analysis of quantitative scales such as the EQ-5D-3L or the confidence level will be performed with matched (or otherwise Wilcoxon) Student tests between inclusion and M12. Random multivariate linear models will be implemented to adjust for any confounding variables. For the EQ-5D-3L, analyses of time until deterioration can be implemented if necessary.

#### Degree of Statistical Significance

The results will be considered significant at the 5% threshold.

#### Methods for Considering Missing, Unused, or Invalid Data

No imputation of missing values will be performed.

### Ethical Considerations

Declarations indicating that the research will be conducted in accordance with the protocol and good practice and legislative and regulatory provisions are in force. The protocol is in accordance with the principles of ethics established by the 18th World Medical Assembly (Helsinki 1964) and the amendments established at the 29th (Tokyo 1975), 35th (Venice 1983), 41st (Hong Kong 1989), 48th (Somerset West 1996), 52nd (Edinburg 2000), 54th (Washington 2002), and 59th World Medical Assembly (Seoul 2008) and reviewed at the 64th World Medical Assembly (Fortaleza 2013). The protocol will be conducted in accordance with the International Conference of Harmonization guidelines of Good Clinical Practice.

## Results

This study was supported by a grant from the French Ministry of Health (PHRIP PARACT 2016-0405) and the Lucien Neuwirth Institute of Cancerology of Saint-Etienne, France. Patients’ data collection for this study has been ongoing since 2018.

## Discussion

The purpose of this project is to implement the PAM-13 within oncology nursing practices at 5 comprehensive cancer centers. To our knowledge, no measurement of patient activation has been used in routine practice or has been experimented in cancerology in France. Analyzing the impact of nursing intervention through such a measure, depending on the type of facility, could lead to strengthening some of them, modifying care practices, and enabling better patient management. In the end, this evaluation via the PAM-13 should allow for a greater understanding of the contribution of these specific nursing interventions of care, the objective being to confirm better activation of the patient in establishments that use a combined set of measures.

The use of an activation measure would constitute a major change in the management of patients with cancer. As the patient’s activation is evolutive, the implementation of targeted nursing interventions would increase the activation. A number of programs have demonstrated their ability to increase the level of patient activation [85-87]. They generally focus on the acquisition of new skills by patients and support a sense of ownership of their health. These different research studies showed that peer support, changes in the patient’s social environment, coaching, and health education courses have been deemed valuable [85-87]. Furthermore, measuring the level of activation and the implementation of measures intended to increase the level of activation would constitute a significant advantage in reducing social health inequalities [88]. Indeed, different studies having implemented this type of intervention have shown that the patients who experience the lowest activation levels are those who tend to show the greatest increase in the activation level [46]. This may be partially due to a “ceiling” effect; not much improvement can be made in patients who already demonstrate high levels of activation. However, this also shows that effective interventions can help disengaged patients become proactive toward their health [85,89,90].

Finally, we assume that studying the implementation of a tool for measuring the degree of activation of the patient of nursing practices in oncology would enable a dynamic evaluation of changes in the patient’s cancer care pathway in order to optimize the latter. The implementation methodology using the RE-AIM evaluation framework would specify the real-life efficiency of the PAM-13, the different adaptations of the centers that have implemented it routinely, the key success factors and pitfalls to avoid, and the various parameters impacting its efficiency [66]. Due to the study and its resulting detailed and analytical description of the contents of the use of the PAM-13 in the comprehensive cancer center concerned, we will produce recommendations for the dissemination of such an intervention in order to manage the deployment of an activation measurement for all new patients with cancer at any requesting facility.

In conclusion, this study of implementation conducted at 5 French comprehensive cancer centers could help define a new strategy for sustaining patients’ empowerment in the cancer care pathway. The use of a PAM would constitute a notable change in the management of patients with cancer. Systematic integration of the measure should make it possible to implement targeted oncology nursing interventions, strengthening some of them in order to offer patients a personalized cancer care pathway adapted to their needs. Indeed, with the arrival of immunotherapy combinations, it appears essential to focus on patients’ empowerment in the management of their medical care. Moreover, the measurement of the level of activation and the implementation of measures to strengthen such activation would constitute a significant advantage in reducing social health inequalities.

## Appendix

Multimedia Appendix 1 Overview of the implementation outcomes from the RE-AIM (Reach, Effectiveness, Adoption, Implementation, and Maintenance) framework.

Multimedia Appendix 2 SPIRIT checklist.

Multimedia Appendix 3 Peer-review reports from DGOS-France, translated in English.

## Abbreviations

COM-B: Capability, Opportunity, Motivation, and Behavior
EQ-5D-3L: European Quality of Life-5 Dimensions-3 Level
HAD: Hospital Anxiety and Depression
PAM-13: Patient Activation Measure-13 item
PARACT: PARAmedical Interventions on Patient ACTivation
RE-AIM: Reach, Effectiveness, Adoption, Implementation, and Maintenance
REALM-R: rapid estimate of adult literacy in medicine-revised
TDF: Theoretical Domains Framework
