# Supplementary material for: Effect of PARACT (PARAmedical Interventions on Patient ACTivation) on the Cancer Care Pathway: Protocol for Implementation of the Patient Activation Measure-13 Item (PAM-13) Version
Source: JMIR Res Protoc. 2020 Dec 8;9(12):e17485. doi: 10.2196/17485 (PMC7755534; doi:10.2196/17485)
Supplement: Multimedia Appendix 1 [file resprot_v9i12e17485_app1.docx]

Table 1. Overview of the Implementation outcomes from the RE-AIM

| **Construct** | **Methods of measurement** |
| --- | --- |
| **Population Reached (R)** | **Adoption by the main parties involved**: Number of participating medical and auxiliary personnel, number of patients monitored by each nursing staff member, initial training of medical and auxiliary staff members  **Representativeness:** Compatibility of the included patient population with the target population (demographic characteristics: Are the oldest patients, those who live a considerable distance away from the hospital and/or the most vulnerable patients interested in monitoring their activation?)  **Participation**: Number of patients who agreed to be monitored, number of patients who were offered follow-up |
| **Effectiveness of the intervention (E)** | **- Effectiveness in real life**   - Measurement of the level of activation of patients and of nursing devices influencing it via the PAM-13 validated in French, will be conducted at the stages of enrollment, 6 and 12 months - Measurement of patients’ level of anxiety: The Hospital Anxiety and Depression scale (HAD) will be used to measure the evolution of patients’ anxiety at enrollment, at 6 and 12 months. - Measurement of patient quality of life: a general quality of life questionnaire EQ-5D 3L will be proposed to patients at enrollment, at 6 and 12 months. - Measurement of the level of health literacy will be assessed at enrollment, 6 and 12 months via the REALM-R, validated in French.   **- Effectiveness of practices**   - Number of patients engaged in monitoring their activation - Proportion of patients completing all proposed monitoring - Assessment of the impact of the PAM-13 measurement: number of patients for whom an intervention of any kind was stimulated, following the measurement of the level of activation - Assessment of the impact of REALM-R measurement: number of patients for whom an intervention of any kind was stimulated, following the measurement of the activation level - The illness, and, depending on the benefit for specific features of the intervention age, stage or progression of the disease and/or complications - Number of patients who participated in at least one PAM-13 assessment during the 12 months - Number of patients who participated in at least one REALM-R assessment during the 12 months   **- Evaluations**   - Patient satisfaction via a questionnaire - Nurses’ satisfaction   **- Context assessment employing mixed methods**   - Qualitative assessment - Quantitative assessment: the sociodemographic characteristics of the patients, the cultural characteristics and characteristics related to the care practices of each institution (organizational characteristics, health professionals, geographical characteristics) will be collected - Factors influencing the level of activation of patients will be studied: type of intervention proposed, type of intervention chosen, sociodemographic characteristics of the patient, characteristics of the cancer, patient complexities, misguided or erroneous use of healthcare system   **- Other criteria according to the recommendations issued at the National Patient Navigation Leadership Summit (NPNLS):**   - Diagnostic confirmation time - Time between diagnosis and management by the facility - Time to first treatment initiation - Number of contacts with the facility - Diagnostic stage - European Deprivation Index (EDI)   **- Analytical statistics on interaction tests will be produced**   - to identify the interaction between contextual factors - to determine the effectiveness of nursing devices in terms of patient activation |
| **Adoption & Adaptation of the intervention to its context (A) and Implementation (I)** | **Precision:** collection of all details and tools necessary for the practical implementation of the PAM-13 according to the establishments: identification of the "body" of the tool for measuring patient activation common to the establishments  **Expertise of health personnel:** Collection of the training level of each medical staff member or healthcare worker  **Adaptation:** comparison of modifications made according to the establishments |
| **Maintenance of the Intervention (M)** | **Assessment of the sustainability of the intervention by identifying the means for institutionalizing the PAM-13 and REALM-R measurement tools:**   - Automated integration into the patient cancer care pathway - Organization of the process - Formalization of the process - Financial facilitators - Administrative recognition |
