# Supplementary material for: Effect of PARACT (PARAmedical Interventions on Patient ACTivation) on the Cancer Care Pathway: Protocol for Implementation of the Patient Activation Measure-13 Item (PAM-13) Version
Source: JMIR Res Protoc. 2020 Dec 8;9(12):e17485. doi: 10.2196/17485 (PMC7755534; doi:10.2196/17485)
Supplement: Multimedia Appendix 3 [file resprot_v9i12e17485_app3.pdf]

PHRIP-16-0405 – expertise 01
Translated version

Utility and Innovation
1. The announced results of the project will be a scientific input AND should allow publication in an international audience journal.
Very Agree
2. The announced results of the project will have a direct impact on the patient's health (mortality, morbidity or quality of life) OR/AND on their management strategy.
Very Agree
3. The announced results of the project, translated into standard practice, represent progress in patient management (mortality, morbidity or quality of life) OR/AND an impact, in individual terms, on the strategy of their management.
Very Agree
4. The announced results of the project, translated into common practice, will have a collective impact on the effectiveness of patient management.
Completely Agree

Investigation approach
1. The state of the art of the field covered by the project, the assumptions and the primary and secondary objectives resulting therefrom are described AND are linked in a consistent manner.
Very Agree
2.The primary and secondary endpoints correspond to the primary and secondary objectives AND are accurately described
Very Agree
3.Methods for measuring the primary and secondary endpoints are described in the project.
Somewhat Agree
4.The selection criteria (inclusion, non-inclusion and exclusion) of the research subjects are described AND consistent with the target project population.
Completely Agree
5.Investigation methods are described and adequate.
Somewhat Agree
6.The experimental plan is adapted AND linked to the question asked.
Very Agree
7.The number of subjects to be included is estimated from the primary endpoint using an appropriate method AND based on justified computational assumptions.
Somewhat Agree
8.The recruitment capacity of participating centres is presented, justified and adequate, including in relation to the project schedule.
Completely Agree
9.The coordinating team's expertise in the project area is documented.
Somewhat Agree
10.The coordinating team and structures they use have the ability to complete the project
Completely Agree
11.The announced resources mobilised and requested enable the project to be completed
Very Agree
12.Ethically, the project may cause you problems AND/OR its implementation may cause problems.
Somewhat disagree with this statement

General assessment of the project
Relevant project answering a current and poorly assessed question in France. The methods of the investigation are described and adequate, however the expertise of the coordinating team is insufficiently documented. The number of subjects to be enrolled is consistent with the data for the primary endpoint, however the method of calculation is not described. In the submitted dossier, the methods for measuring the number of serious adverse events are not detailed (even if in the case at hand the study does not include any invasive investigations or investigations that could generate major adverse events).

PHRIP-16-0405– expertise 02
Translated version

Utility and Innovation
1. The announced results of the project will be a scientific input AND should allow publication in an international audience journal.
Somewhat Agree
2. The announced results of the project will have a direct impact on the patient's health (mortality, morbidity or quality of life) OR/AND on their management strategy.
Completely Agree
3. The announced results of the project, translated into standard practice, represent progress in patient management (mortality, morbidity or quality of life) OR/AND an impact, in individual terms, on the strategy of their management.
Completely Agree
4. The announced results of the project, translated into common practice, will have a collective impact on the effectiveness of patient management.
Completely Agree

Investigation approach
1. The state of the art of the field covered by the project, the assumptions and the primary and secondary objectives resulting therefrom are described AND are linked in a consistent manner.
Completely Agree
2.The primary and secondary endpoints correspond to the primary and secondary objectives AND are accurately described
Completely Agree
3.Methods for measuring the primary and secondary endpoints are described in the project.
Completely Agree
4.The selection criteria (inclusion, non-inclusion and exclusion) of the research subjects are described AND consistent with the target project population.
Completely Agree
5.Investigation methods are described and adequate.
Completely Agree
6.The experimental plan is adapted AND linked to the question asked.
Completely Agree
7.The number of subjects to be included is estimated from the primary endpoint using an appropriate method AND based on justified computational assumptions.
Completely Agree
8.The recruitment capacity of participating centres is presented, justified and adequate, including in relation to the project schedule.
Completely Agree
9.The coordinating team's expertise in the project area is documented.
Completely Agree
10.The coordinating team and structures they use have the ability to complete the project
Completely Agree
11.The announced resources mobilized and requested enable the project to be completed
Completely Agree
12.Ethically, the project may cause you problems AND/OR its implementation may cause problems.
Somewhat disagree with this statement

General assessment of the project

This project seems to be able to meet the objectives it sets itself, namely to objectify the results of paramedical actions with regard to the resources mobilized in four care structures. It can provide quality indicators of care and professional practices, and therefore contribute to their improvement. It is part of a scientific approach aimed at promoting paramedical practices and producing evidence on their effectiveness. The theme and the health problem addressed are fully integrated, in several respects, into public health priorities (fight against cancer, care for chronic diseases, care for ageing and fragile populations, fight against social inequalities in health).
